# Development of RNA-FISH Assay for Detection of Oncogenic *FGFR3-TACC3* Fusion Genes in FFPE Samples

**DOI:** 10.1371/journal.pone.0165109

**Published:** 2016-12-08

**Authors:** Masahiro Kurobe, Takahiro Kojima, Kouichi Nishimura, Shuya Kandori, Takashi Kawahara, Takayuki Yoshino, Satoshi Ueno, Yuichi Iizumi, Koji Mitsuzuka, Yoichi Arai, Hiroshi Tsuruta, Tomonori Habuchi, Takashi Kobayashi, Yoshiyuki Matsui, Osamu Ogawa, Mikio Sugimoto, Yoshiyuki Kakehi, Yoshiyuki Nagumo, Masakazu Tsutsumi, Takehiro Oikawa, Koji Kikuchi, Hiroyuki Nishiyama

**Affiliations:** 1 Department of Urology, Faculty of Medicine, University of Tsukuba, Tsukuba, Japan; 2 Astellas Pharma Inc., Tsukuba, Japan; 3 Tsukuba Clinical Research and Development Organization (T-CReDO), Faculty of Medicine, University of Tsukuba, Tsukuba, Japan; 4 Department of Urology, Graduate School of Medicine, Tohoku University, Sendai, Japan; 5 Department of Urology, Graduate School of Medicine, Akita University, Akita, Japan; 6 Department of Urology, Graduate School of Medicine, Kyoto University, Kyoto, Japan; 7 Department of Urology, Faculty of Medicine, Kagawa University, Kagawa, Japan; 8 Department of Urology, Hitachi General Hospital, Hitachi, Japan; 9 Department of Urology, Tsukuba Medical Center Hospital, Tsukuba, Japan; Universita degli Studi di Torino, ITALY

## Abstract

**Introduction and Objectives:**

Oncogenic *FGFR3-TACC3* fusions and *FGFR3* mutations are target candidates for small molecule inhibitors in bladder cancer (BC). Because *FGFR3* and *TACC3* genes are located very closely on chromosome 4p16.3, detection of the fusion by DNA-FISH (fluorescent in situ hybridization) is not a feasible option. In this study, we developed a novel RNA-FISH assay using branched DNA probe to detect *FGFR3-TACC3* fusions in formaldehyde-fixed paraffin-embedded (FFPE) human BC samples.

**Materials and Methods:**

The RNA-FISH assay was developed and validated using a mouse xenograft model with human BC cell lines. Next, we assessed the consistency of the RNA-FISH assay using 104 human BC samples. In this study, primary BC tissues were stored as frozen and FFPE tissues. *FGFR3-TACC3* fusions were independently detected in FFPE sections by the RNA-FISH assay and in frozen tissues by RT-PCR. We also analyzed the presence of *FGFR3* mutations by targeted sequencing of genomic DNA extracted from deparaffinized FFPE sections.

**Results:**

*FGFR3-TACC3* fusion transcripts were identified by RNA-FISH and RT-PCR in mouse xenograft FFPE tissues using the human BC cell lines RT112 and RT4. These cell lines have been reported to be fusion-positive. Signals for *FGFR3-TACC3* fusions by RNA-FISH were positive in 2/60 (3%) of non-muscle-invasive BC (NMIBC) and 2/44 (5%) muscle-invasive BC (MIBC) patients. The results of RT-PCR of all 104 patients were identical to those of RNA-FISH. *FGFR3* mutations were detected in 27/60 (45%) NMIBC and 8/44 (18%) MIBC patients. Except for one NMIBC patient, *FGFR3* mutation and *FGFR3-TACC3* fusion were mutually exclusive.

**Conclusions:**

We developed an RNA-FISH assay for detection of the *FGFR3-TACC3* fusion in FFPE samples of human BC tissues. Screening for not only *FGFR3* mutations, but also for *FGFR3-TACC3* fusion transcripts has the potential to identify additional patients that can be treated with FGFR inhibitors.

## Introduction

Activation of *fibroblast growth factor receptor 3* (*FGFR3*) has been reported to play important roles in several malignancies, including uterine cervix carcinoma and multiple myeloma as well as bladder cancers (BC)[1][2]. Ninety-seven percent of activating *FGFR3* mutations observed in BC are clustered in either exon 7 (codons 248 and 249), exon 10 (codons 372, 373 and 375), or exon 15 (codon 652)[3]. Mutations in exons 7 or 10 create unpaired cysteines in the proximal extracellular region, leading to the formation of disulfide bonds between adjacent receptors, thereby inducing ligand-independent dimerization and activation[4][5]. Mutations within the kinase domain, such as codon 652, are thought to induce a conformational change in the activation loop, resulting in constitutive autophosphorylation of the receptor[6].

Recently, *FGFR3-transforming acidic coiled-coil 3* (*TACC3*) fusions have been identified in glioblastoma[7], head and neck carcinoma[8], and lung cancer[9], as well as urothelial cancer (UC)[10]. FGFR tyrosine kinase inhibitors have been developed and shown to be effective in cell lines harboring not only an activating *FGFR3* mutations, but also an *FGFR3-TACC3* fusion gene in vitro and in vivo[11]. These include the S249C mutation in human BC cells 97–7[12], Y375C mutation in human BC cells MGH-U3[13], and *FGFR3-TACC3* fusion in human glioma stem cells GIC-1123[11]. In addition, significant clinical responses to an FGFR inhibitor were reported in *FGFR3-TACC3* fusion-positive patients with cervical cancer[14] or glioma[11] in Phase I clinical trials. Thus, detection of not only the activating *FGFR3* mutations, especially in exons 7, 10 and 15, but also the *FGFR3-TACC3* fusion in BC patients could be clinically important to identify responders to FGFR kinase inhibitors.

DNA fluorescent in situ hybridization (DNA-FISH) is widely used to detect fusion genes from genomic DNA[15][16]. However, genomic DNA-FISH is not a feasible option to detect an *FGFR3-TACC3* fusion. Generally, fusion detection assays of DNA-FISH are based on 2 strategies, dual fusion or break apart. In the dual fusion strategy, 2 colored probes are designed to span the breakpoint of the 2 genes involved in the fusion. These probes are visually distinct in normal cells but appear merged by the specific fusion event. However, this strategy is not a feasible option for *FGFR3-TACC3* fusion detection because the 2 genes map very closely, at a distance of only 48 Kb on chromosome 4p16.3, and thus the 2 probes appear merged in both normal cells and fusion-positive cells. In the break-apart strategy, probes are designed to target opposite sides of the translocation break point for a given gene, each labeled by a different color. These probes generate signals in normal cells that are co-localized and appear merged. Following a translocation, the signals are no longer co-localized but appear to be separate. This strategy is also not a feasible option for *FGFR3-TACC3* fusion detection. According to Parker et al., the *FGFR3-TACC3* fusion is caused by tandem duplication of a 70 kb region on 4p16.3[17]. This is confirmed by performing genomic DNA capillary sequencing of tandem duplication boundaries[17]. Therefore, fluorescent probes appear merged in both normal and fusion-positive cells.

Recently, several RNA-ISH based assays have been applied to detect mRNA transcripts of fusion genes, which include padlock probes/rolling circle amplification (RCA), “smFISH,” and “branched DNA (bDNA)-FISH”. For example, *TMPRSS2-ERG* fusion transcripts were identified by padlock probes/RCA[18]. However, the padlock probe/RCA approach needs an in situ cDNA preparation, which would pose additional technical difficulty when using formaldehyde-fixed paraffin-embedded (FFPE) tissue slides due to degradation and modification of nucleic acids. “smFISH” systems using single fluorophore-labeled probes are also applied to detect mRNA transcripts of fusion genes[19][20]. Following smFISH, “bDNA-FISH” system has been applied to detect mRNA transcripts of fusion genes[21]. In “bDNA-FISH,” sequential hybridization of a series of oligonucleotide probes generates signal amplification. This contrasts with “smFISH,” which lacks a signal-amplification step. The bDNA probes are commercially available as a 'ViewRNA' system (Affymetrix, Santa Clara, CA, USA) or an 'RNAscope' system (Advanced Cell Diagnostics, Hayward, CA, USA). “bDNA-FISH” and “smFISH” demonstrate the same accuracy, but “bDNA-FISH” yields brighter spots with a better signal-to-noise ratio[22]. Neither “smFISH” nor “bDNA-FISH” assay has been applied to detect *FGFR3-TACC3* fusion yet.

Here, we sought to develop an RNA-FISH assay using bDNA probes to detect *FGFR3-TACC3* fusion gene transcripts in FFPE tissue, which are the most widely available specimens in clinical settings. In this study, we first applied an RNA-FISH assay using bDNA probes to detect *FGFR3-TACC3* fusion transcripts in human FFPE BC tissue. We also analyzed the relationship between the *FGFR3* mutation, *FGFR3-TACC3* fusion status, and clinical information in a prospective multicenter cohort of more than 100 patients with the clinical diagnosis of BC.

## Materials and Methods

### Patients and tissue samples

This study was conducted as a prospective multicenter cohort study including 144 patients from 7 institutions with the clinical diagnosis of UC. The participating hospitals were Tohoku University Hospital, Akita University Hospital, Kyoto University Hospital, Kagawa University Hospital, Hitachi General Hospital, Tsukuba Medical Center Hospital, and Tsukuba University Hospital. Primary cancer tissue samples obtained from 106 patients with non-metastatic BCs were stored as frozen and FFPE tissues. The remaining 38 cases were metastatic UC includes bladder, ureter, and renal pelvis cancer. For these metastatic UC patients, archival FFPE samples of the primary tumors were used if fresh frozen tissues were not available.

This research protocol was approved by the Ethics Committee of Tsukuba University Hospital (Approval number: H25-116). This study was also reviewed and approved by the Ethics Committees of the following institutes: Tohoku University Hospital, Akita University Hospital, Kyoto University Hospital, Kagawa University Hospital, Hitachi General Hospital, and Tsukuba Medical Center Hospital. Tumor specimens, blood, and clinicopathologic information were collected with written informed consent.

Portions of tissue samples were frozen and stored at −80°C, and the remainder of the sample was fixed in 10% formaldehyde for 12–24 hours at room temperature and embedded in paraffin for diagnostic assessment. Hematoxylin and eosin staining (H&E) was performed, and the slides were reviewed by a pathologist. Tumors were staged according to the 2009 UICC 7th TNM Classification system. Tissue sections that showed malignant tumor cell nuclei in 10% or more cells on the whole specimen were included in this study. A total of 144 patients were enrolled, 7 patients were excluded from this study due to a low tumor fraction in the whole specimen (less than 10%). Two patients were excluded because their tumors were not malignant. One sample was excluded due to a lack of genomic DNA yield from the FFPE sample. Thirty samples were excluded because fresh frozen tissues were not available. Finally, 104 BC patients were included. The patient characteristics are summarized in Table 1. Of the 104 patients, 60 were classified as non-muscle invasive BC (NMIBC) and 44 as muscle-invasive BC (MIBC) according to the pathological findings.

**Table 1:** Characteristics of bladder cancer patients

		NMIBC	MIBC	Total
N		60	44	104
Age (years)	Median (range)	69 (30–87)	72 (42–87)	70 (30–87)
Gender	Male (%)	55 (92)	32 (73)	87 (84)
	Female (%)	5 (8)	12 (27)	17 (16)
T stage	Ta (%)	43 (72)		
	Tis/T1 (%)	17 (28)		
	≧T2 (%)		44 (100)	
M stage	M1 (%)	0 (0)	4 (10)	
Grade	High grade (%)	26 (47)		
	Low grade (%)	34 (53)		
Multiplicity	Solitary (%)	24 (40)	23 (52)	
	Multiple (%)	33 (55)	15 (34)	
	Unknown (%)	3 (5)	6 (14)	
Tumor size	<3 cm (%)	41 (68)	9 (20)	
	>3 cm (%)	19 (32)	35 (80)	

### Cell lines and cell culture

Three human BC cell lines (RT112[23], *FGFR3-TACC3* fusion-positive[10]; RT4[24], *FGFR3-TACC3* fusion-positive[10]; and T24[25], *FGFR3* wild type[10]) and HSC-39 (human signet ring cell gastric carcinoma cell line[26], *FGFR3* wild type) were used. All cells were cultured in RPMI 1640 (Wako, Osaka, Japan) supplemented with 10% fetal bovine serum (FBS) at 37°C in 5% CO2 and 20% O2.

### Subcutaneous xenografts and FFPE slide preparation

For establishment of tumor xenografts, log-phase RT112, RT4, T24, and HSC-39 cells were implanted intradermally (5×10^6^ cells per mouse in 0.1 mL PBS) into the backs of female BALB/c-nu/nu mice (Charles River Laboratories, Wilmington, MA, USA) at 5 to 6 weeks old. All surgery was performed under ether anesthesia, and all efforts were made to minimize suffering. Mice were euthanized by cervical dislocation while under anesthesia when tumor size exceeded 200 mm^3^ in size. Tumors were excised from mice and divided into 2 pieces. One piece was dip-washed in saline, blotted dry, snap frozen in liquid nitrogen, and stored at −80°C until analysis. The other piece was fixed in 10% formaldehyde for 24 hours at room temperature before embedding in paraffin for tissue sections. All experiments were performed in compliance with the relevant Japanese and institutional laws and guidelines and approved by the University of Tsukuba Animal Ethics Committee (authorization number 15–162).

### Detection of fusion transcripts by RNA-FISH

In situ detection of *FGFR3* and *TACC3* transcripts in FFPE sections was conducted using a QuantiGene® ViewRNA ISH Tissue Assay Kit (Affymetrix) with a modified protocol for custom-made probes. For *TACC3*, Alexa 546 (Excitation = 556 nm and Emission = 573 nm) was used, and for *FGFR3*, Alexa 647 (Excitation = 650 nm and Emission = 668 nm) was used as a fluorescent dye; both were purchased as custom-made products (namely type 1 for *TACC3* and type 6 for *FGFR3*) from Veritas (an Affymetrix sales representative in Japan). A step-by-step protocol is provided as the S1 Protocol. In brief, FFPE sections were treated according to the manufacturer’s protocol of QuantiGene® ViewRNA ISH Tissue Assay Kit before adding the labeled probe solution. In order to detect the mRNA signal with high resolution, the fluorescent label reagent (label probe mix) from a QuantiGene ViewRNA ISH Cell Assay Kit (Affymetrix) and its compatible custom-made probes described above were used.

**Fig 1:**
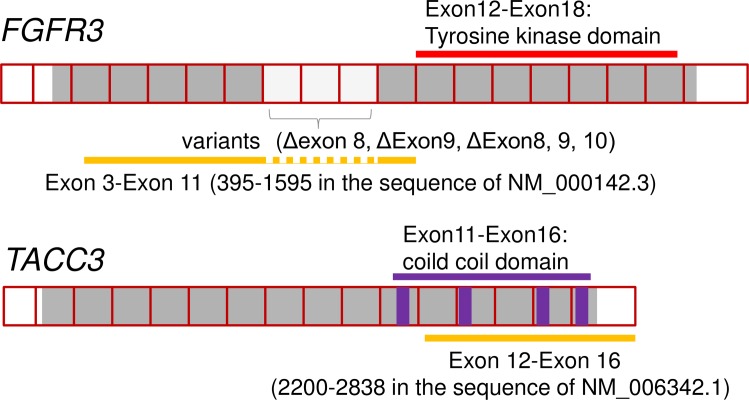
Schematic representation of how *FGFR3*-specific probes and *TACC3*-specific probes were designed.

**Fig 2:**
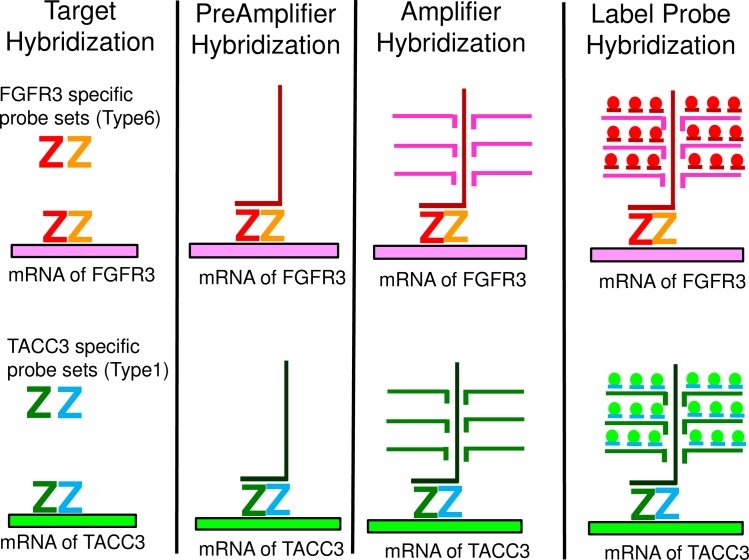
A schematic figure explaining how bDNA-FISH works.

The target sequence of the *FGFR3*-specific oligonucleotide probes was located in exons 3 to 11 corresponding to nucleotide numbers 395–1595 in the sequence of NM_000142.3 of *FGFR3* except for exons 8, 9 and 10, which are variable in alternative splicing. Thus the probes specifically hybridize to all three variants of human *FGFR3* mRNA. The target sequence of the *TACC3*-specific oligonucleotide probes was located in exons 12–16 corresponding to the nucleotide numbers 2200–2838 in the sequence of NM_006342.1 of *TACC3*. Fig 1 shows a schematic representation of how *FGFR3*-specific probes and *TACC3*-specific probes were designed. The probe sets for each gene consisted of around 15 to 20 pairs of probes. One pair consisted of 2 of about 20 base oligonucleotide probes. The 2 probe pairs are designed to hybridize to adjacent segments on the target RNA, allowing further hybridization of a preamplification probe (Affymetrix) for signal amplification by labeled probes. This “double Z” structure assures the specificity to the target mRNA because this construction of a “double Z” structure by 2 adjacent probes is required for signal amplification. A schematic figure explaining how this bDNA-FISH-based assay works is shown in Fig 2.

After fluorescent label probe hybridization, slides were dried and mounted with ProLong® Gold Antifade Reagents (Thermo Fisher Scientific, Waltham, MA, USA). Ten non-overlapping fields of view of fluorescent signal images per slide were obtained for each fluoresce with ×630-fold magnification with a confocal laser microscope (LSM700; Carl Zeiss, Zena, Germany). For *FGFR*, a Cy5 (Excitation/Emission = 650/670 nm) filter was used, and for *TACC3*, a Cy3 (Excitation/Emission = 552/570 nm) filter was used. Each field of view was obtained from a different part of the tissue portion in which cancer cells were present. The presence of cancer cells was confirmed by comparing DAPI-stained nucleic images with H&E stained images. These images were analyzed using IN Cell Investigator Developer Toolbox 1.9.2 which is the image analysis software used with IN Cell Analyzer 2000 (GE Healthcare, Amersham, UK) to obtain the number of signals for each probe (*FGFR3* and *TACC3*) and to examine whether each signal dot from each probe overlapped or not. Overlapped/co-localized dots were recognized as “overlapped/co-localized” when 50% of the area from each signal overlapped with another signal. When overlapped/co-localized signals were observed, the number of such overlapped/co-localized dots were counted. These image analyses were performed unbiasedly and automatically by software with a fixed protocol every time. A schematic representation of how co-localized signals were detected by IN Cell Investigator is shown in S1 Fig. The overlapped signals could be detected not only from real fusion mRNA, but also from proximity of the 2 target RNAs simply by chance. Actually, a small number of overlapped signals were detected in the negative control HSC-39. These accidentally overlapped signals could be increased in direct proportion to the total number of *FGFR3* signals and *TACC3* signals. To overcome this problem, the number of overlapped signals was divided by the number of *FGFR3* and *TACC3* signals. These processes were repeated for each 10 non-overlapping fields of view per one sample. The co-localization ratios were defined as the ratio of the co-localized count number per total number for each probes, and this co-localization ratio for 10 non-overlapping fields in each slide was plotted as in a scatter diagram and used for detection of the presence of fusion transcripts.

### RNA extraction and cDNA synthesis

Total RNA was extracted from frozen tissues using TRIzol (Thermo Fisher Scientific), and from FFPE tissues using an RNeasy FFPE kit (Qiagen, Hilden, Germany). First strand cDNA was synthesized using Superscript Ⅲ Super Mix and oligo dT primers (Thermo Fisher Scientific) according to the manufacturer’s instructions. The quality of the cDNA from FFPE tissue was tested for the presence of the *hypoxanthine guanine phosphoribosyltransferase* (*HPRT*) housekeeping gene (152 bps amplified product; forward primer, 5’-GACTTTGCTTTCCTTGGTC-3’ and reverse primer, 5’-AGTCAAGGGCATATCCTAC-3’). The quality of cDNA from frozen tissue was tested for the *β-actin* housekeeping gene (539 bps amplified product using forward primer 5’-GTGGGGCGCCCCAGGCACCA-3’ and reverse primer 5’-CTCCTTAATGTCACGCACGATTTC-3’).

### Identification of *FGFR3-TACC3* fusion transcripts by RT-PCR and DNA sequencing

**Fig 3:**
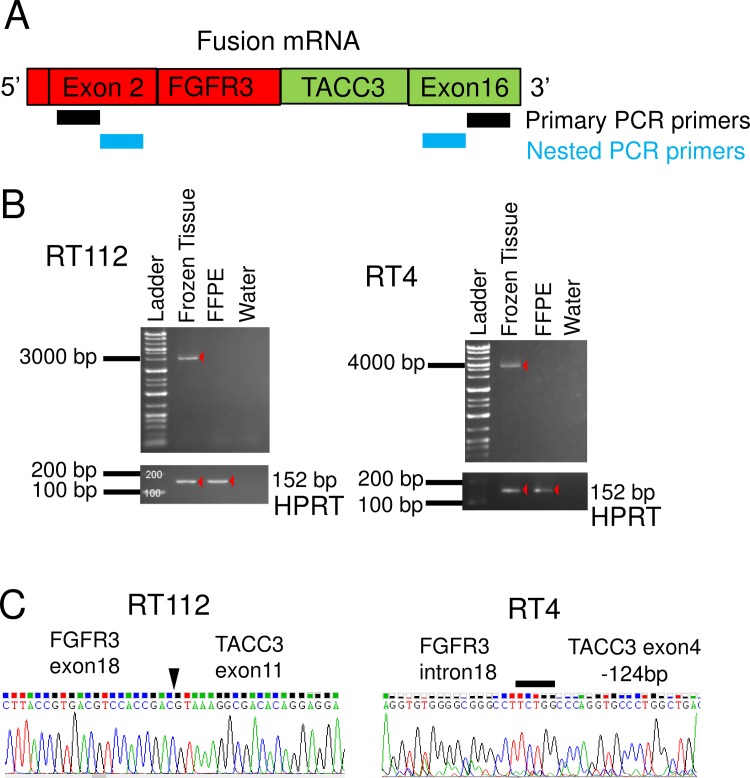
*FGFR3-TACC3* fusion transcript detection by RT-PCR. (A) Schematic representation of *FGFR3-TACC3* fusion mRNA and PCR primers position. (B) Agarose gel separation of the *FGFR3-TACC3* fusion specific RT-PCR amplicons. (C) Sanger sequencing chromatogram of *FGFR3-TACC3* fusion specific RT-PCR products. The arrowhead and solid bar indicate breakdown point or region of the 2 genes.

We designed a reverse transcription-PCR (RT-PCR) assay for the detection of all known and possible new variants of *FGFR3-TACC3* fusions that retain the mRNA sequences coding for the key FGFR-tyrosine kinase domain and transforming acidic coiled-coil domain required for oncogenic activity of the fusion protein. Previously reported *FGFR3-TACC3* fusions were exons 18 or 19 of *FGFR3* and exons 4–13 of *TACC3*, and a short intron was inserted in some cases[27]. To detect *FGFR3-TACC3* fusion gene transcripts, we performed RT-PCR using our original primers as follows: the forward primer, *FGFR3* exon2-Forward: 5’- CCTGAGGACGCCGCGGCCCCCGCCCCC-3’ and the reverse primer, *TACC3* exon16-Reverse: 5’-TGACCTCCACGGAGCCGCTGTCCCCGC-3’; amplification conditions were 94°C for 2 min (98°C for 5 sec/68°C for 3 min) for 40 cycles, then 72°C for 5 min. PCR-amplified products were diluted 1:50 in sterile water, and then used for nested PCR amplifications with internal primers. For the nested PCR, primer pairs were *FGFR3* exon 2-Forward: 5’- GCCATGGGCGCCCCTGCCTGCGCCCTC-3’ and *TACC3* exon 16-Reverse: 5’- GACCTCATCTCCAAGATGGAGAAGATC-3’. A schematic representation of RT-PCR primer positions is shown in Fig 3A. All designed primers were BLASTed against the human genome to make sure they were not complementary to other regions of the genome[28]. After amplification of the fusion gene specific template by PCR, the finding was confirmed by agarose gel electrophoresis according to the expected length of the amplicon (RT112; 2850 bps and RT4; 4461 bps). The PCR products obtained as described above were purified and sequenced by the Sanger method. Conventional fluorescent dye chemistry sequencing was performed on an ABI Prism 3130xl Genetic Analyzer (Applied Biosystems, Foster City, CA, USA) according to the manufacturer’s instructions.

### Mutation analysis

Genomic DNA was extracted using a QIAamp DNA FFPE Tissue Kit (Qiagen) according to the manufacturer’s instructions. The DNA concentration for each sample was assessed by a Qubit fluorometer (Thermo Fisher Scientific). Genomic DNA with more than 1.5 ng/μL according to the Qubit fluorometer was subjected to further mutation analysis. The presence of mutations in *FGFR3* was analyzed by targeted sequencing using Ion AmpliSeq Cancer Hotspot Panel v2 (Thermo Fisher Scientific). The targeted *FGFR3* mutations are listed in S1 Table. For data analysis, Torrent Suite 4.0.2 was used, and mutations were detected by the Variant Caller plugin 4.0–6 with somatic/high stringency configuration provided by Ion Torrent (Thermo Fisher Scientific).

### Statistical analysis

Differences among groups were analyzed using Fisher’s exact test. P values of <0.05 were considered to be statistically significant. Statistical analyses were performed using Jmp11 software (SAS Institute Inc., Cary, NC, USA).

## Results

### Identification of *FGFR3-TACC3* fusion transcripts by sequencing and RNA-FISH from xenograft FFPE tissue using human bladder cancer cell lines

**Fig 4:**
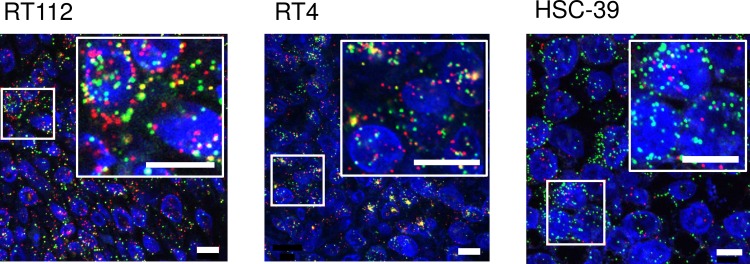
*FGFR3-TACC3* fusion transcript detection by RNA-FISH. RNA-FISH image of RT112 and RT4 (fusion-positive controls) and HSC-39 (negative control) xenograft FFPE tissue. mRNAs of *FGFR3* and *TACC3* were detected by RNA-ISH using fluorescent probes (Alexa 647 for *FGFR3* and Alexa 546 for *TACC3*, respectively), and signals from *FGFR3* and *TACC3* were shown as red and green, respectively, in the figure. The small boxed areas are enlarged in the adjacent large boxes. Fusion mRNAs appeared in microscope images as yellow, which are merged signals from red and green colors. Cell nuclei was stained with DAPI and shown as blue. Scale bars in the figure are 10 μm. For the detection of fusion signals for each sample, image data from 2 fluorescent probes were analyzed with IN Cell Analyzer 2000 and the number of overlapped/co-localized signals was counted and divided by the total number of *FGFR3* signals and *TACC3* signals and plotted in a scatter graph.

To develop methods to detect *FGFR3-TACC3* fusion transcripts, we first tried RT-PCR methods using mouse xenograft models of the human BC cell lines RT112 and RT4. Both cell lines were reported[10] to harbor *FGFR3-TACC3* fusion genes. Xenograft tissues were divided into 2 fractions; one was stored frozen, and the other was stored as FFPE tissue. The amplified products of the *HPRT* housekeeping gene (152 bps) were identified in FFPE samples as well as in frozen tissues. PCR products of *FGFR3-TACC3* fusion genes were found at about 2800 bps and 4500 bps in frozen tissues from xenografts of RT112 and RT4, respectively, but not in FFPE samples (Fig 3B). Sequencing of PCR products confirmed that both cell lines harbored the same break-point sequence as previously reported[10] (Fig 3C). Next, we tried RNA-FISH to detect *FGFR3* and *TACC3* signal using xenograft FFPE tissues of RT112 (fusion-positive control) and RT4 (fusion-positive control) and HSC-39 cells (fusion-negative control). Fluorescent signals for *FGFR3* and *TACC3* were detected in FFPE samples of all three xenografts. Overlapped/co-localized signals were abundant in xenograft FFPE sections with RT112 and RT4, but barely detected in HSC-39 cells (Fig 4).

### Detection of *FGFR3-TACC3* fusion genes from clinical FFPE samples by RNA-FISH

**Fig 5:**
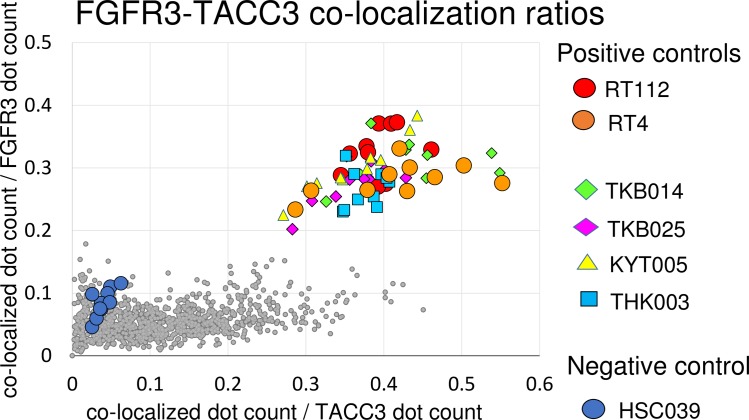
Detection of *FGFR3-TACC3* fusion genes in FFPE clinical samples by RNA-FISH. Scatter diagram of *FGFR3-TACC3* co-localization ratios of 10 non-overlapping fields for each sample. The number of co-localized signals was divided by the number of *FGFR3* signals and *TACC3* signals, and the quotients were plotted in Y- and X-axis, respectively. Four samples in the right upper quadrant were thought to be fusion positive by RNA-FISH, and were confirmed as fusion positive by RT-PCR analysis. Small gray dots represent cases that were thought to be negative for the fusion gene.

**Fig 6:**
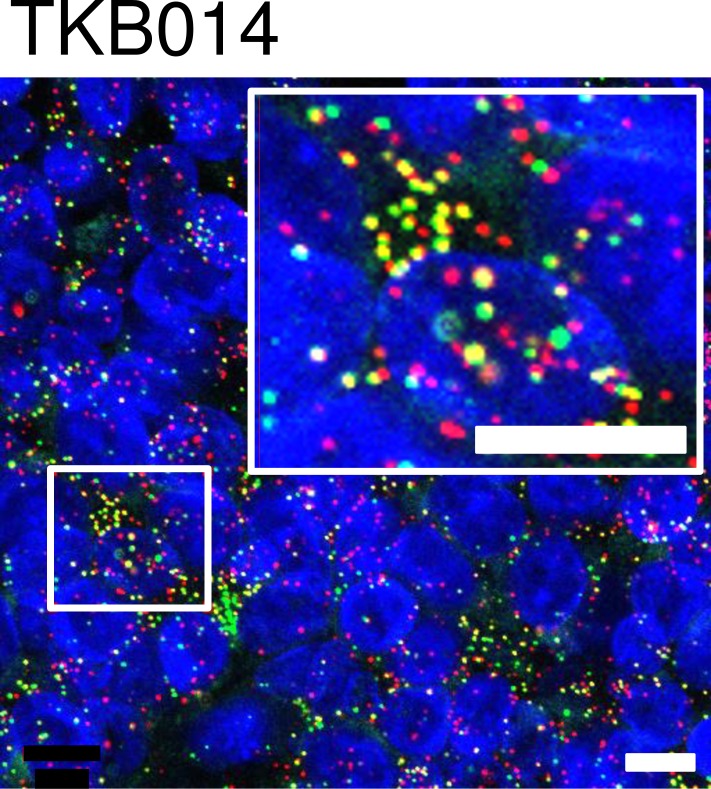
Representative RNA-FISH images of fusion-positive case TKB014. The small boxed areas are enlarged in the adjacent large boxes. Scale bars in the figure are 10 μm.

**Fig 7:**
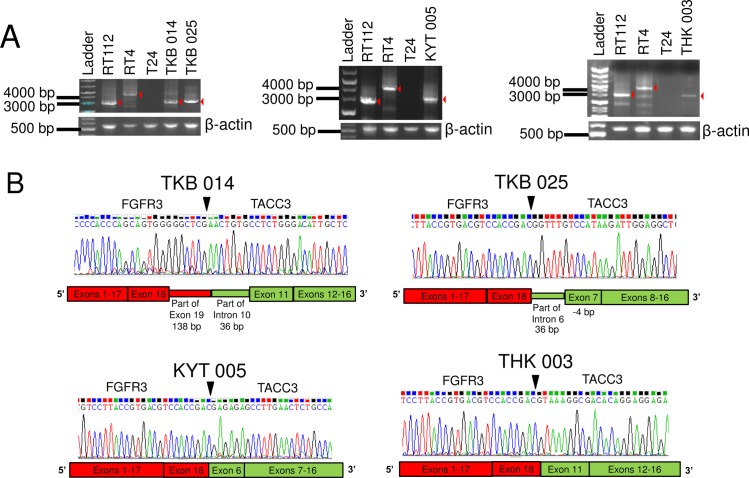
Detection of *FGFR3-TACC3* fusion transcripts in clinical samples by RT-PCR. (A) Agarose gel separation of the *FGFR3-TACC3* fusion-specific RT-PCR amplicons. (B) Sanger sequencing chromatogram of *FGFR3-TACC3* fusion-specific RT-PCR products. Arrowheads indicate breakdown points of the 2 genes.

To examine whether RNA-FISH is applicable to human samples, we analyzed 104 BC samples independently by RNA-FISH and Sanger sequencing to detect *FGFR3-TACC3* fusions as a prospective cohort study. Tumor samples were freshly collected, and parts of the samples were fixed in 10% formaldehyde for 12–24 hours at room temperature and then embedded in paraffin. For detection of the fusion transcript by RNA-FISH, the number of overlapped/co-localized signals was divided by the number of *FGFR3* signals and *TACC3* signals, and the quotients were plotted in a scatter graph (Fig 5). The original result of signal count analysis by IN Cell Analyzer 2000 are described in S2 Table. Four of 104 samples and positive controls are plotted in the right upper quadrant (both ratios of co-localized dot count/FGFR3 dot count and co-localized dot count/TACC3 dot count are greater than 0.2). These 4 cases were thought to be fusion positive. A representative photomicrograph of RNA-FISH in the *FGFR3-TACC3* fusion-positive sample TKB014 is shown in Fig 6. Small gray dots in Fig 5 represent the cases that were thought to be negative for the fusion gene. Fluorescent signals were very weak in 2 cases (KYT004 and TMC001), so these cases were suspended from fusion analysis by RNA-FISH result. To assess the sensitivity and specificity of RNA-FISH, frozen tissues from all 104 BC were tested using RT-PCR methods. RT-PCR products around 2800–4500 base pairs were identified in all 4 cases with positive signals for fusion genes in RNA-FISH (Fig 7A). Sanger sequencing demonstrated that these 4 cases indeed had *FGFR3-TACC3* fusion genes with different break-point sequences (Fig 7B). Two cases were NMIBC, and the remaining 2 cases were MIBC. Positive rates of *FGFR3-TACC3* fusion were 2/60 (3%) and 2/44 (5%) in NMIBC and MIBC patients, respectively. The other 98 patients were fusion negative with both PCR and RNA-FISH. The 2 suspended cases of FISH analysis (KYT004 and TMC001) were determined as fusion negative by the RT-PCR result.

### Relationship between *FGFR3* mutation, *FGFR3-TACC3* fusion, and clinical information

**Fig 8:**
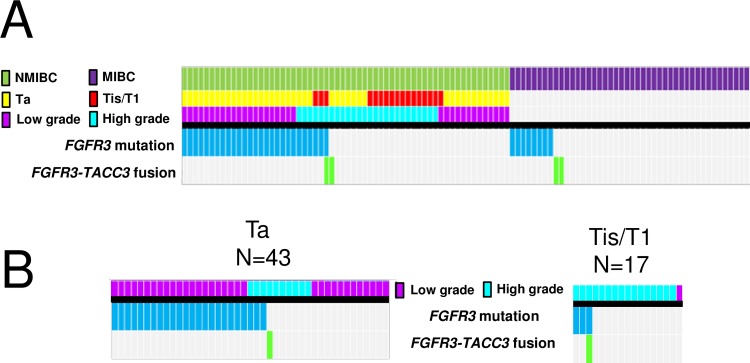
*FGFR3* mutation and *FGFR3-TACC3* fusion status. (A) The heatmap shows the distribution of *FGFR3* mutations and *FGFR3-TACC3* fusions with respect to T stage and pathological grade. (B) Subgroup analysis of NMIBC by T stage.

*FGFR3* hotspot mutations were detected in 27/60 (45%) and 8/44 (18%) of NMIBC and MIBC cases, respectively (Fig 8A). No mutation was detected in *FGFR3* coding regions in 3 of the 4 patients with an *FGFR3-TACC3* fusion. One case (TKB025) with an *FGFR3-TACC3* fusion gene also had a concomitant mutation in *FGFR3* (S249C). In this case, the *FGFR3*-S249C mutation was detected from tumor FFPE tissue-derived genomic DNA by Ion AmpliSeq Cancer Hotspot Panel v2. However, the corresponding codon in the full-length *FGFR3-TACC3* fusion transcript was not altered.

Among NMIBC cases, *FGFR3* mutation/fusion were more frequent (Fig 8A) in low-grade tumors (21/34; 62%) than in high-grade tumors (7/26; 27%) (*P* = 0.0066). In a subgroup analysis of NMIBC tumors by T stage, *FGFR3* mutations/fusions were more frequent in Ta tumors (25/43, 58%) than in T1 tumors (3/16, 19%) (*P* = 0.034) (Fig 8B). The presence of an *FGFR3* mutation/fusion was not associated with tumor size or tumor multiplicity in either NMIBC or MIBC patients.

## Discussion

Generally, fusion genes are detected by immunohistochemistry (IHC), FISH, and RT-PCR. IHC assays have demonstrated a wide variation in sensitivity and specificity in FFPE tissues[29]. Specimen processing, antigen retrieval, and immunodetection systems have significant effects on the results of IHC staining[30]. In addition, positive IHC staining does not represent the chimeric fusion protein itself but the expression level of one of the fusion partner genes. In glioblastoma, possible associations between IHC staining of FGFR3 protein and the presence of *FGFR3-TACC3* fusion were reported[11][17]. In the present study, 75% of *FGFR3-TACC3* fusion-positive tumors showed a high level of FGFR3 protein staining (S1 Appendix). Further studies are needed to confirm this association. In addition, the FGFR3 protein expression level and *FGFR3* mutation status were found to be significantly associated[31][32], but 15–26% of *FGFR3* mutant tumors showed low levels of FGFR3 expression by IHC. In the present study, 12% of *FGFR3* mutant tumors showed a low level of FGFR3 protein staining (S1 Appendix). These cases could be missed if IHC is used as a screening tool for the detection of patients who bear an *FGFR3* mutation or *FGFR3-TACC3* fusion.

As mentioned in the introduction, genomic DNA-FISH is not a feasible option to detect *FGFR3-TACC3* fusion. RNA-FISH is another option to detect fusion transcripts from FFPE tissue. There are several technologies that can be used to detect mRNA transcripts, which include padlock probes/RCA, “smFISH,” and “bDNA-FISH,” as summarized in a previous article[33]. Femino et al. were the first to describe the visualization of single RNA molecules by FISH[34]. Raj et al. improved the original single-molecule RNA FISH (smFISH) using short oligos each labeled with a single fluorophore[35]. “smFISH” was applied by Semrau et al. (FuseFISH)[20] and Markey et al. (Fusion FISH)[19] to detect oncogenic fusion transcripts. In “bDNA-FISH,” multiple pairs of primary probes are required to hybridize in a juxtaposed position (double Z design) in order to proceed with branched DNA hybridization for signal amplification (Fig 2). This contributes increased specificity and reduced background signal. This method is commercially available as a QuantiGene® VeiwRNA kit (Affymetrix) or RNAScope® kit (Advanced Cell Diagnostics). Battich et al. reported that “smFISH” and “bDNA-FISH” demonstrate the same accuracy, but that bDNA-FISH yielded brighter spots with a better signal-to-noise ratio(22).

Here we used this bDNA-FISH-based “QuantiGene® ViewRNA ISH Tissue Assay Kit” to detect fusion mRNA. This assay is suitable for not only fresh tissue, but also archival FFPE tissues. Urdinguio et al. assessed 124 FFPE human colon cancer tissues that had been stored more than 5 years. They confirmed that *GM-CSF* (*granulocyte-macrophage colony-stimulating factor*) mRNA was overexpressed in human colorectal cancer using this assay[36]. Weier et al. evaluated the expression level of ETV4 and ETV5 in 83 FFPE human prostate cancer tissue samples that had been stored 8 to 19 years[37]. In this study, we developed an RNA-FISH assay using bDNA probe to detect *FGFR3-TACC3* fusion and assessed its sensitivity and specificity using FFPE human samples. The results of RNA-FISH using FFPE sections were identical to those obtained by RT-PCR using frozen tissues, indicating that RNA-FISH is a feasible assay for screening for *FGFR3-TACC3* fusion. Our data suggested that an RNA-FISH for fusion genes might be feasible for screening other fusion genes when genomic DNA-FISH lacks utility.

RT-PCR was thought to be another option to detect *FGFR3-TACC3* fusion transcripts in FFPE tissue. To detect all known and possibly new variants of *FGFR3-TACC3* fusions, the forward primer needs to recognize a sequence upstream of exon 18 of the *FGFR3* and the reverse primer downstream of exon 13 of the *TACC3*. The size of the PCR amplicon using this primer pair varies by case from between 100 to more than 1,000 base pairs. RNA obtained from FFPE tissues is generally highly degraded, making it difficult to detect a PCR amplicon of several hundred base pairs or more. Even if we design a multiplex PCR primer to cover to all known combinations of exons on the *FGFR3* and *TACC3*, the expected length of the PCR amplicon varies by case for the following reasons. First, the genomic break point could be different in each case. Second, introns could be inserted between the genomic break point of the *FGFR3* and *TACC3* genes in some cases[27]. Actually, 36 bps of intron 10 of the *TACC3* gene were inserted into fusion mRNA in TKB014 in our study. Thus, the expected lengths of PCR amplicons of the ready-made multiplex PCR primer for each exon were unequal and predisposed to give a false negative. Recently, targeted sequencing using next-generation sequencing was applied for detection of fusion genes. Ross et al. applied targeted sequencing to FFPE tissues of 35 UC patients, and the *FGFR3-TACC3* fusion was detected in one patient[38]. Targeted sequencing might be an option to detect fusion genes when fusion partners are already known.

In previous studies, the frequencies of *FGFR3* mutations were 60–80% of the low-grade NMIBC and 5–20% of the invasive tumors[2][39][40][41][42], which is consistent with the results of the present study. As reported in many previous studies[43][44], the proportion of cases having an *FGFR3* mutation decreased with increasing stage and grade. In this study, *FGFR3-TACC3* fusion genes were present in 2/60 (3%) of NMIBC and 2/44 (5%) of MIBC cases. In previous studies, the fusion was detected in 6% (1/17) of NMIBC cases[45] and 2–4% (3/129[46], 2/46[10], 1/25[45], and 1/35[38]) of MIBC cases. These results are consistent with the present study. Both *FGFR3-TACC3* fusion and *FGFR3*-S249C mutation were positive in case TKB025. The full-length *FGFR3-TACC3* fusion transcript in this case did not involve a *FGFR3*-S249C mutation. This result may indicate the presence of tumor heterogeneity of a wildtype codon 249, fusion-positive clone and codon 249 mutant, fusion negative clone. In another study, 2 different *FGFR3* mutations were detected in a single case[47]. The association between the presence of an *FGFR3-TACC3* fusion gene and the prognosis of UC is still unclear. We plan to follow the subjects of this prospective study with detailed clinical information for three years. In the future, we hope we can provide additional evidence.

The selection of an optimal drug is determined by the genetic profile of some cancers, including lung cancer, breast cancer, and leukemia. Patient selection by molecular foundation is not yet indicated for metastatic UC. Significant clinical responses were reported in *FGFR3-TACC3* fusion-positive cervical cancer patients and glioma patients treated with an FGFR inhibitor[11][14]. However, the *FGFR3* mutation is not very frequent among metastatic UC patients, comprising about 6–18% (2/35[38], 2/11[47], and 3/33[48]). The screening of not only the *FGFR3* mutation, but also the *FGFR3-TACC3* fusion gene makes it possible to find additional responders to FGFR inhibitors.

A limitation of this method is that it cannot detect fusions other than *FGFR3-TACC3*. *BAIAP2L1*(*BAI1-associated protein 2-like 1*) was reported as another fusion partner of *FGFR3* in lung cancer patients[49] and BC cell lines[10]. The fusion gene leads to a constitutive activation of the *FGFR3* tyrosine kinase domain. An *FGFR3-BAIAP2L1* fusion-positive cell line showed sensitivity to an FGFR inhibitor in vitro[50]. In this study, the *FGFR3-BAIAP2L1* fusion gene was not examined. The association between the *FGFR3-BAIAP2L1* fusion and clinical stages of UC is still unclear. The importance of the detection for this fusion will be determined by further investigation of its frequency in BC. The *BAIAP2L1* gene is located on chromosome 7 at position 7q22.1, which is a different chromosome from the location of the *FGFR3* gene. The *FGFR3-BAIAP2L1* fusion has been detected by DNA-FISH, which is easily applied to clinical cases[10][50].

## Conclusions

In this study, we applied an RNA-FISH assay to detect *FGFR3-TACC3* fusion transcripts in 104 FFPE BC tissues. We demonstrated that RNA-FISH is a feasible assay to screen for the *FGFR3-TACC3* fusion. We also analyzed the association of *FGFR3* mutation/fusion status and clinical information in a prospective multicenter cohort of 104 patients with a clinical diagnosis of BC. The efficiency of the bDNA-FISH assay needs to be confirmed in a larger series of cases and possibly compared with the smFISH methodology that has also been reported to be an accurate approach for the detection of fusion transcripts, before it could be considered the technique of choice to identify additional responders to FGFR inhibitors.

## Supporting Information

S1 Appendix FGFR3 Protein Expression Analysis in Tissue Samples Using Tissue Microarrays (DOCX)

S1 Fig A schematic representation of how co-localized signals were detected by IN Cell Investigator Developer Toolbox 1.9.2. (TIF)

S2 Fig Low-power view of one of the tissue microarrays with FGFR3 immunostaining. Scale bar = 5 mm.
(TIF)

S3 Fig Patterns of FGFR3 staining intensity. (A) Staining pattern 0; (B) staining pattern 1; (C) staining pattern 2; (D) staining pattern 3. Scale bars = 100 μm.
(TIF)

S4 Fig Comparison of FGFR3 staining intensity with FGFR3 mutation and FGFR3-TACC3 fusion status. Dark blue bars = High, Light blue bars = Low.
(TIF)

S1 Protocol A step-by-step protocol for RNA FISH using a ViewRNA kit. (DOCX)

S1 Table The list of targeted FGFR3 mutations for Ion AmpliSeq Cancer Hotspot Panel v2. The original excel file is available at: http://tools.invitrogen.com/downloads/cms_106003.csv
(XLSX)

S2 Table The result of RNA-FISH image analysis by IN Cell Analyzer 2000. (XLSX)

S3 Table FGFR3 staining intensity and *FGFR3* mutation/*FGFR3-TACC3* fusion status. (XLSX)
