# Supplementary material for: Development of RNA-FISH Assay for Detection of Oncogenic FGFR3-TACC3 Fusion Genes in FFPE Samples
Source: PLoS One. 2016 Dec 8;11(12):e0165109. doi: 10.1371/journal.pone.0165109 (PMC5145148; doi:10.1371/journal.pone.0165109)
Supplement: S1 Appendix — (DOCX) [file pone.0165109.s001.docx]

**FGFR3 Protein Expression Analysis in Tissue Samples Using Tissue Microarrays**

A total 103/104 cases of FFPE cancer tissues were used. The FFPE tissue block was not available for one case (pT1a, Low grade, *FGFR3* mutant). After carefully choosing the morphologically representative region on the individual paraffin-embedded blocks (donor blocks), two or three representative tumor-bearing core cylinders (diameter 2 mm) were obtained from each donor paraffin block and transferred into the recipient paraffin block. Successful transfer of tumor tissue was confirmed microscopically using Hematoxylin-Eosin-stained sections. In summary, the TMA contained 59 NMIBC, 44 MIBC.

Immunohistochemical (IHC) staining for FGFR3 protein (Monoclonal, #4574; Cell Signaling, Danvers, MA, USA) was performed according to the manufacturer's instructions. Briefly, Endogenous peroxidase was blocked with 3% hydrogen peroxidase for 10 min. Antigen retrieval was done by heat slides in a sub-boiling temperature (95°-98°C) citric acid buffer for 10 minutes using microwave oven. Then, sections were incubated overnight with primary antibodies (dilution 1:50) at 4℃. A subsequent reaction was performed with the VECTASTAIN Elite ABC kit, including biotinylated secondary antibody (Vector Laboratories, Burlingame, CA, USA). Antibody specificity was confirmed using sections of tumor obtained from human bladder cancer cell line xenograft. The FGFR3 RNA expression level of these cell lines were previously assessed by real-time RT–PCR (human bladder cancer cell line RT112 ; high, and T24; low) [12]. 　Evaluation of the IHC results was performed independently by two investigators (MK, TKawahara) blinded to all patient data. Differences were settled by consensus following review of individual cases. FGFR3 staining intensity was assessed according to a semi-quantitative scoring system reported by Tomlinson et al.[32]: 0, all tumor cells negative; 1, faint but detectable positivity in some or all cells; 2, weak but extensive positivity; 3, strong positivity (regardless of extent). If cores obtained from one specimen showed different scores, the highest expression level was considered.

**RESULTS**

Immunohistochemistry was successfully carried out on 103 samples (S2 and S3 Figs, S3 Table). There was a high level of agreement between scorers (81%, 83/103). Grouping low or normal expression patterns (0 and 1) into a ‘low’ category and higher expression patterns (score 2 and 3) into a ‘high’ category, the concordance between scorers increased to 85% (88/103). Overall, 88% (29/33) of *FGFR3* mutant tumors showed over-expression, and 75% (3/4) of fusion positive tumors showed over-expression (S4 Fig). By contrast, only 20% (13/66) of *FGFR3* wild-type tumors showed over-expression.
