# Supplementary figures and images for: Development of RNA-FISH Assay for Detection of Oncogenic FGFR3-TACC3 Fusion Genes in FFPE Samples
Source: PLoS One. 2016 Dec 8;11(12):e0165109. doi: 10.1371/journal.pone.0165109 (PMC5145148; doi:10.1371/journal.pone.0165109)

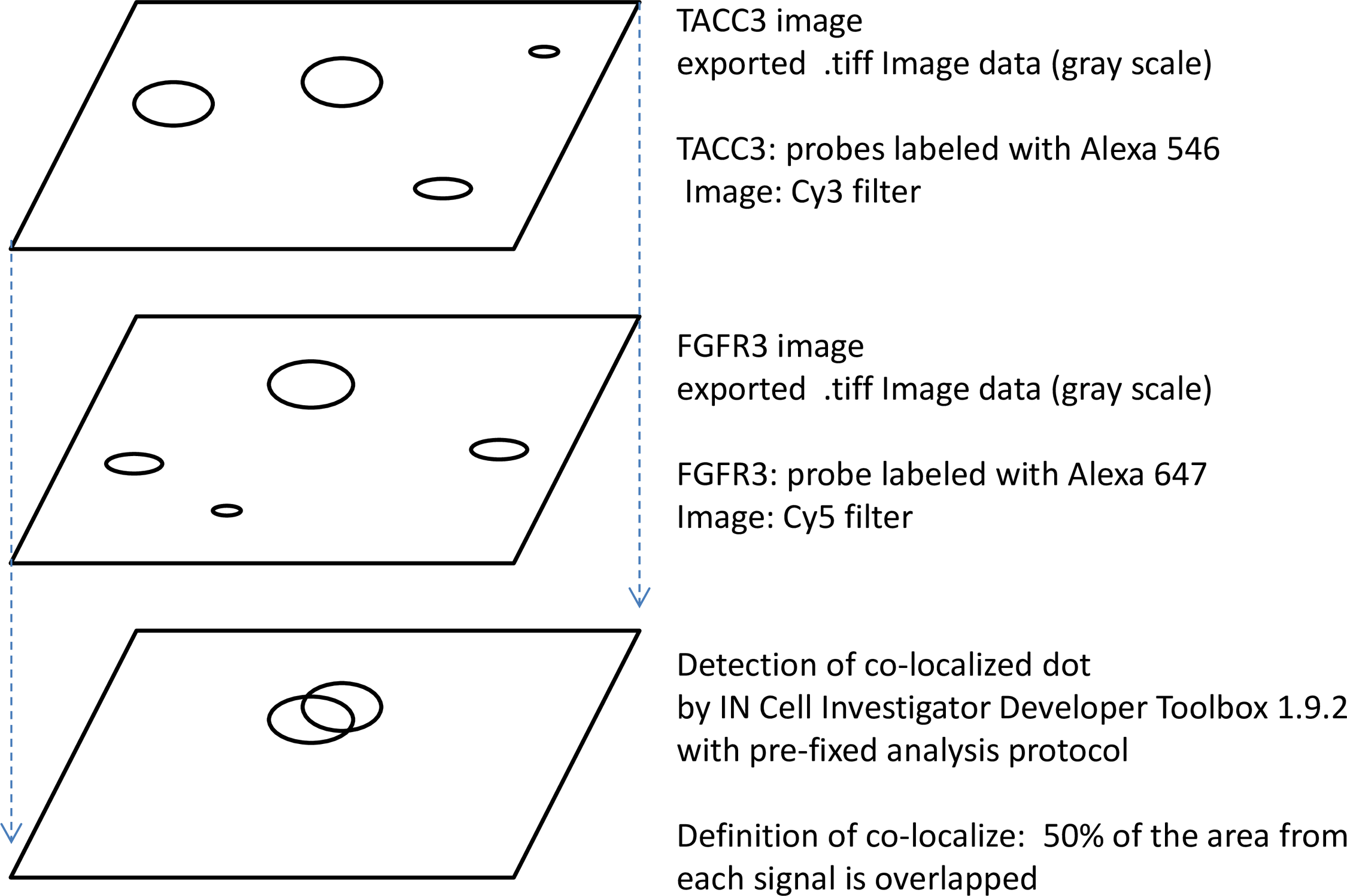

Supplement: S1 Fig — (TIF) [file pone.0165109.s002.tif]

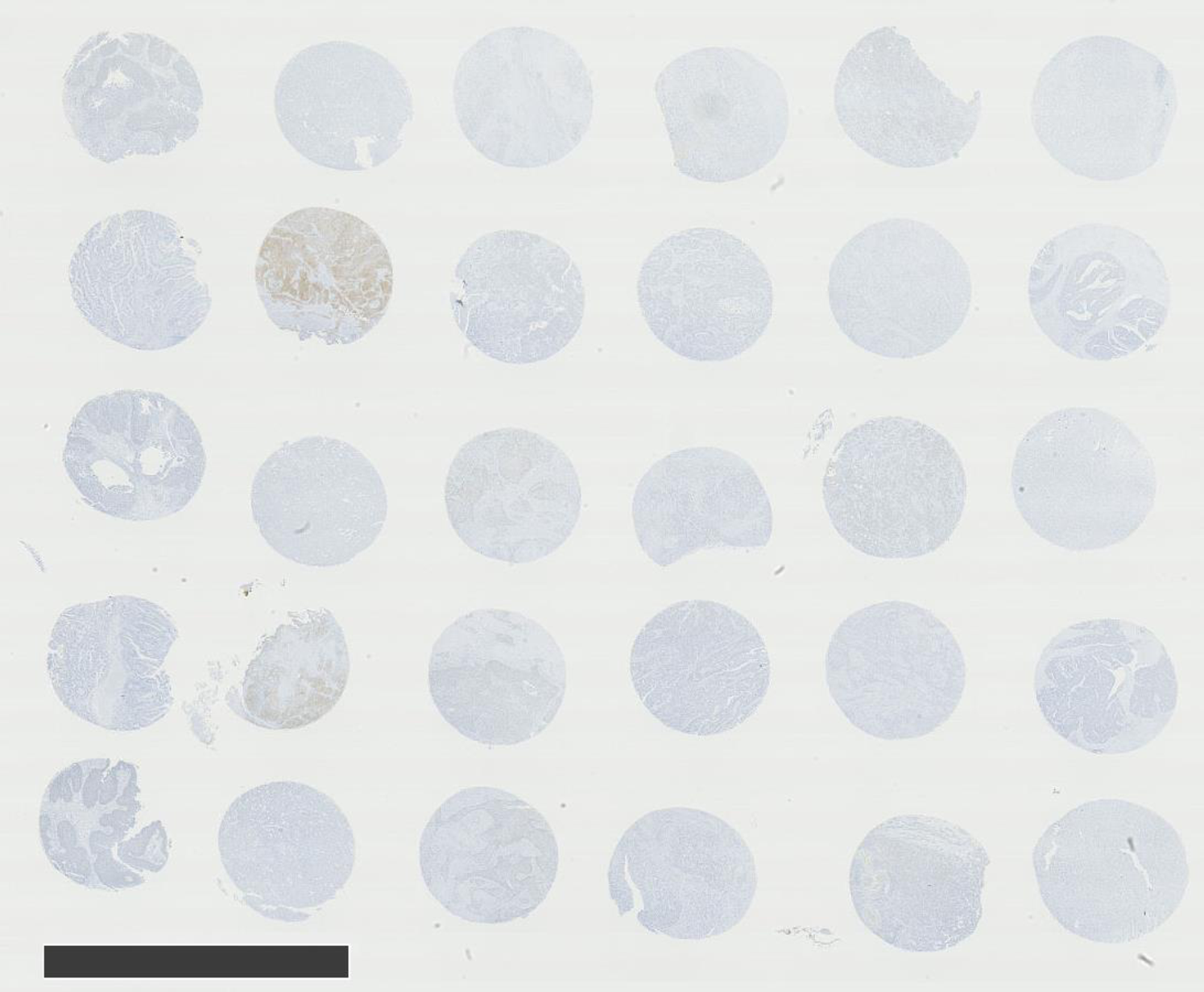

Supplement: S2 Fig — Scale bar = 5 mm. (TIF) [file pone.0165109.s003.tif]

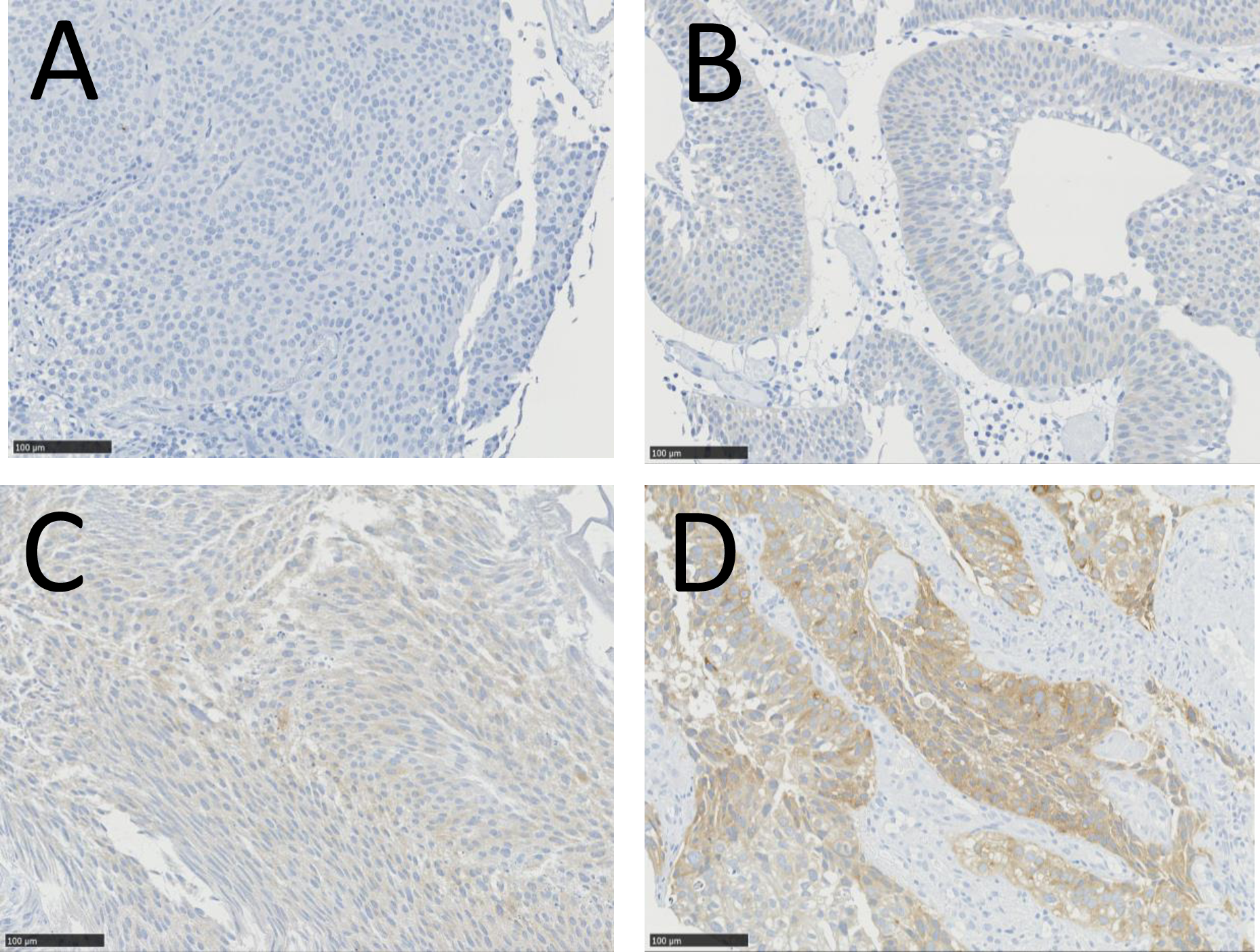

Supplement: S3 Fig — (A) Staining pattern 0; (B) staining pattern 1; (C) staining pattern 2; (D) staining pattern 3. Scale bars = 100 μm. (TIF) [file pone.0165109.s004.tif]

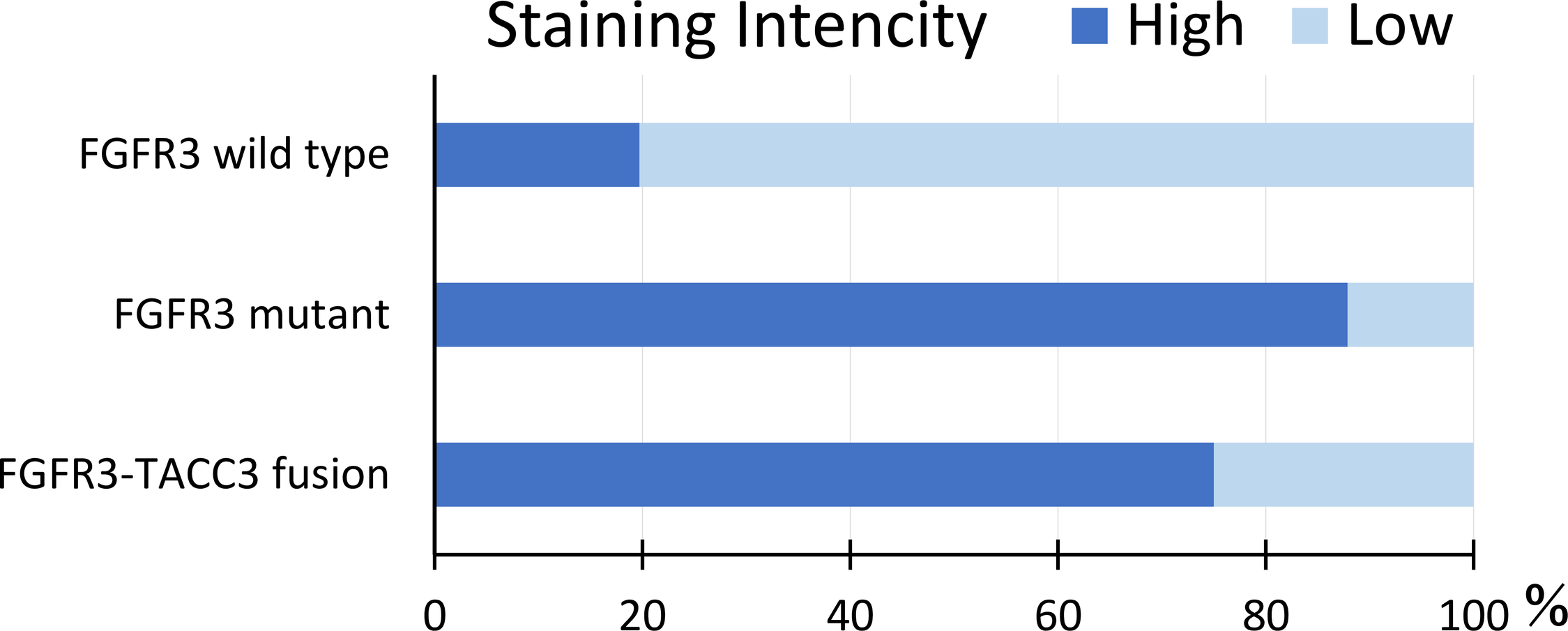

Supplement: S4 Fig — Dark blue bars = High, Light blue bars = Low. (TIF) [file pone.0165109.s005.tif]
