# Supplementary material for: Development of RNA-FISH Assay for Detection of Oncogenic FGFR3-TACC3 Fusion Genes in FFPE Samples
Source: PLoS One. 2016 Dec 8;11(12):e0165109. doi: 10.1371/journal.pone.0165109 (PMC5145148; doi:10.1371/journal.pone.0165109)
Supplement: S1 Protocol — (DOCX) [file pone.0165109.s006.docx]

RNA FISH using ViewRNA kit

- Kit
- QuantiGene® ViewRNA ISH Tissue Assay

(Format: 2-Plex Sample: FFPE Tissue Sections)

- QuantiGene® ViewRNA ISH Cell Assay
- Probes
- Type1 target probe : Human TACC3, #VA1-12748 (VX1-99999-01)

HUMAN TACC3 TYPE 1 (Alexa 546, Ex 556, Em 573, filter:Cy3)

- Type6 target probe : Human FGFR3, #VA6-12749 (VX6-99999-01)

HUMAN FGFR3 TYPE 6 (Alexa 647, Ex 650, Em 665, filter:Cy5)

- RNA ISH procedure
- step (1) to (18) (before adding Label Probe Solution), refer to QuantiGene® ViewRNA ISH Tissue Assay
- steps (19) to (24) (after adding Label Probe Solution), refer to QuantiGene® ViewRNA ISH Cell Assay

1. bake the slides for 15min at 60 ºC
2. fix slides in 10 % formaldehye (29.2 ml 1X PBS and 10.8 ml of 37 % formaldehyde) for 30 min at room temperature
3. wash slides with 40 mL of PBS 3 times, and dry the slides
4. deparaffinization in 40 ml of xylene for 10 min at room temperature
5. wash slides with 40 mL of PBS 3 times
6. tissue pretreatment in boiled Pretreatment Solution (-150 ml) for 10min
7. wash slides with 40 mL of H2O 2 times
8. wash slides with 40 mL of PBS 1 times
9. protease digestion in 300μL of Protease Solution (3μL Protease QF in 297μL PBS (Prewarmed to 40ºC)) for 20 min at 40 ºC
10. wash slides with 40 mL of PBS 3 times
11. fix slides in 40mLof 4% formaldehye for 5 min at room temperature
12. wash slides with 40 mL of PBS 3 times
13. Target probe hybridization in 200μL of Probe Set Solution (5μL TYPE 1 Probe Set, 5μL TYPE 6 Probe Set, in 190μL Probe Set Diluent QT (prewarmed to 40 ºC)) for 2hrs at 40 ºC
14. wash slides with 40 mL of wash buffer 3 times
15. PreAmp hybridization in 200μL of PreAmplifier Mix QT (prewarmed to 40 ºC) for 25 min at 40 ºC
16. wash slides with 40 mL of wash buffer 3 times
17. Amp hybridization in 200μL of Amplifier Mix QT (prewarmed to 40 ºC) for 15 min at 40 ºC
18. wash slides with 40 mL of wash buffer 3 times
19. hybridize with Label Probe Solution (8μL Label Probe Mix in 192μL of Label Probe Diluent QF (prewarmed to 40 ºC)) for 30 min at 40 ºC
20. wash slides with 40 mL of wash buffer 2 times
21. wash slides with 40 mL of PBS
22. DAPI-staining in 300μL of DAPI (3μg/mL in PBS) for 5 min at room temperature
23. wash slides with 40 mL of PBS 2 times
24. Mounting on glass slide using ProLong® Gold Antifade Reagents
